# Closed loop large bowel obstruction due to appendiceal signet cell carcinoma

**DOI:** 10.1093/jscr/rjab452

**Published:** 2021-10-28

**Authors:** Du H Phan, Joshua Y Teo, Sasikaran Nalliah

**Affiliations:** Department of General Surgery, Hervey Bay Hospital, Queensland, Australia; Department of General Surgery, Hervey Bay Hospital, Queensland, Australia; Department of General Surgery, Hervey Bay Hospital, Queensland, Australia

## Abstract

Signet cell carcinoma of the appendix is the rarest and the most aggressive subtype of appendiceal malignancy, typically presenting with non-specific symptoms. We describe a case of a 62-year-old male with large bowel obstruction, with computed tomography demonstrating dilated large bowels from caecum to proximal sigmoid colon and pneumoperitoneum. Intraoperatively, closed loop obstruction caused by dense adherence of sigmoid colon to caecum was noted, which had resulted in caecal perforation. Histopathology study indicated primary appendiceal malignancy of signet cell morphology with intraperitoneal spread to sigmoid colon. Large bowel obstruction from appendiceal malignancy has rarely been reported and similar presentations have not been described in the existing literature. When left-sided large bowel obstruction is suspected to be caused by a malignant stricture, it is essential to consider transperitoneal spread of appendiceal malignancy as potential aetiology, particularly in the elderly.

## INTRODUCTION

Primary appendiceal malignancy is a rare entity and signet cancer is the least common subtype [1]. Due to their predominantly non-specific presentation, preoperative diagnosis of appendiceal malignancy is usually elusive. In this report, we describe an unusual presentation of closed loop large bowel obstruction in a patient with signet cell carcinoma of the appendix.

## CASE REPORT

**Figure 1:**
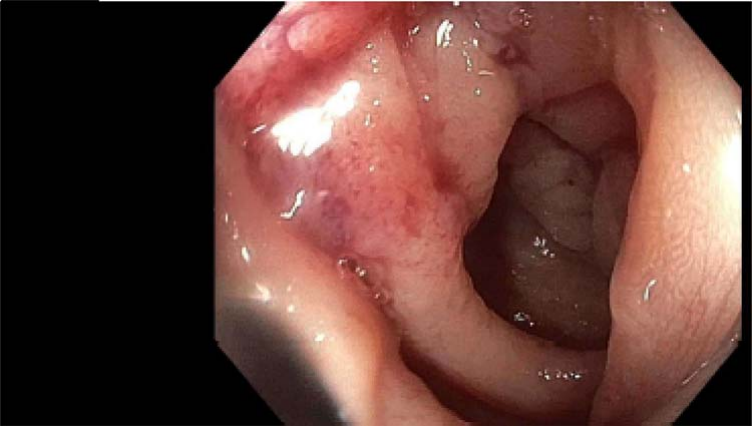
Colonoscopic finding of sigmoid stricture.

A 62-year-old, previously well, male presented to the emergency department with a 2-day history of colicky lower abdominal pain, distension and obstipation. Relevant background included non-insulin-dependent type two diabetes, coronary artery disease and chronic obstructive pulmonary disease. He was noted to have a sigmoid stricture during an outpatient colonoscopy 3 months prior (for polyp surveillance). Although not completely obstructed, the colonoscope could not traverse this stenosis (Fig. 1). The patient was awaiting outpatient computed tomography (CT) colonography to further evaluate this lesion when he presented to the emergency department with obstructive symptoms.

**Figure 2:**
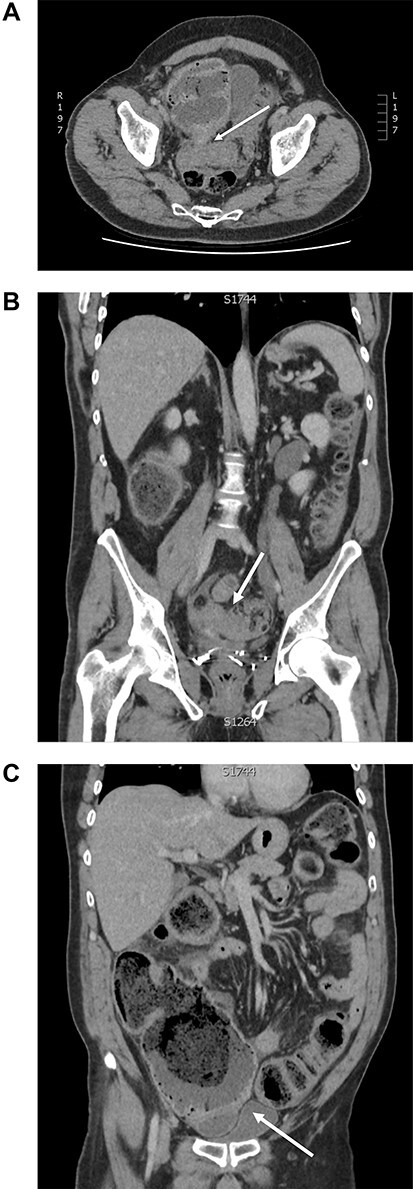
Computed tomography of large bowel obstruction and pneumoperitoneum: (**A**) axial view depicting dilated caecum with pneumatosis and adherence to the sigmoid stricture (white arrow); (B) coronal view depicting sigmoid stricture (white arrow); (C) coronal view depicting pneumoperitoneum, dilated caecum with adhesion to sigmoid colon stricture (white arrow).

At the time of review, patient was febrile (39°C), tachycardic (110 bpm), with four quadrant peritonism and raised inflammatory markers. CT showed distended large bowel loops extending from caecum to a suspected transition point in distal sigmoid colon with pneumoperitoneum (Fig. 2). Emergency laparotomy was arranged for intraabdominal sepsis, presumably from large bowel perforation.

Findings during the laparotomy included a closed loop obstruction of the large bowel with sigmoid stricture densely adherent to the caecum, which could only be separated using blunt finger dissection. Faecal contamination was noted to have arisen from the caecal perforation secondary to closed loop obstruction. In order to manage the dual pathology in the caecum and sigmoid colon, subtotal colectomy with formation of end ileostomy was performed. Histopathology study revealed poorly differentiated carcinoma arising from the appendix with signet ring morphology and transperitoneal extension into sigmoid colon. American Joint Committee on Cancer staging was pT4b pN2 M0 and R1 resection. Immunohistochemistry showed positive staining for CK7, AE1/3, CK20, and was negative for PSA and NXX3.1. Patient has been on adjuvant chemotherapy (FOLFOX regimen) and remains well 6 months post-operation.

## DISCUSSION

Appendiceal malignancy is uncommon, accounting for 0.4–1% of all gastrointestinal malignancies [2]. There are five main subtypes: colonic type adenocarcinoma, mucinous adenocarcinoma, goblet cell carcinoid, malignant carcinoid and signet cell carcinoma. Signet carcinoma is the least common subtype, accounting for about 4% of all appendiceal malignancy [3]. It has the worst prognosis with 5-year survival described as low as 7% [3]. Previous reports have suggested the presence of local invasion and distant metastasis in up to 76 and 93% of patients, respectively, at the time of diagnosis [4, 5].

Preoperative diagnosis of appendiceal malignancy is challenging, due to its non-specific presentation. Previous reports suggested most appendiceal malignancies (37–79.1%) present as appendicitis, whereas others may present as vague abdominal pain or non-specific gastrointestinal symptoms [5–9]. Rarer presentations described included vesico-appendiceal fistula, caeco-colic intussusception, neck mass, per vaginal bleeding, spontaneous entero-cutaneous fistula or bilateral ureteric obstruction. Appendiceal malignancy masquerading as pelvic organ tumours or even as inguinal hernia has also been documented [7].

Appendiceal malignancy presenting with bowel obstruction is uncommon. A few reports have described small bowel obstruction secondary to appendiceal malignancy of goblet cell and signet cell subtypes [1, 10]. To our knowledge, only two case reports have described large bowel obstruction from primary appendiceal cancer [2, 11]. Suzuki *et al*. [11] described signet cell carcinoma with local invasion to the ovaries and sigmoid colon causing large bowel obstruction. Aljarabah *et al*. [2] also described a case of sigmoid colon obstruction secondary to primary appendiceal malignancy, although with adenocarcinoma subtype. No previous report to date has described caecal perforation secondary to closed loop obstruction of the large bowel from appendiceal signet carcinoma. In our case, it is hypothesized that the transperitoneal invasion to the sigmoid colon from the appendiceal malignancy resulted in the sigmoid stricture seen during the elective colonoscopy. The adherence of the caecum and the sigmoid colon served as the ‘choke’ point for closed loop obstruction. This resulted in increased intraluminal pressure of the caecum and eventual perforation. Right hemicolectomy is the preferred surgical intervention for most localized appendiceal malignancy, unless there is clear evidence of peritoneal metastasis intraoperatively. Management of metastatic disease ranges from systemic chemotherapy, hyperthermic intraperitoneal chemotherapy, cytoreductive peritonectomy or combined approach [3]. In the present case, we opted to proceed with subtotal colectomy to manage dual pathology of caecal perforation and sigmoid stricture.

## CONCLUSION

Signet cell carcinoma is the rarest and most aggressive subtype of appendiceal malignancy. Preoperative diagnosis is elusive due to its non-specific presentation. We describe the first report of caecal perforation from large bowel obstruction due to locally invading signet cell carcinoma. Our case contributes to the growing list of non-specific presentations of this elusive malignancy. Although primary sigmoid malignancy is a common cause of closed loop large bowel obstruction in patients with competent ileo-caecal valve, it is pertinent to maintain a high level of suspicion for locally invasive malignancy of appendiceal origin.

## CONFLICT OF INTEREST STATEMENT

None declared.
